# Chagas Cardiomyopathy Presenting as Symptomatic Bradycardia: An Underappreciated Emerging Public Health Problem in the United States

**DOI:** 10.1155/2017/5728742

**Published:** 2017-08-16

**Authors:** Richard Jesse Durrance, Tofura Ullah, Zulekha Atif, William Frumkin, Kaushik Doshi

**Affiliations:** ^1^Department of Internal Medicine, Jamaica Hospital Medical Center, 8900 Van Wyck Expressway, Jamaica, NY 11418, USA; ^2^Department of Cardiology, Jamaica Hospital Medical Center, 8900 Van Wyck Expressway, Jamaica, NY 11418, USA

## Abstract

Chagas cardiomyopathy (CCM) is traditionally considered a disease restricted to areas of endemicity. However, an estimated 300,000 people living in the United States today have CCM, of which its majority is undiagnosed. We present a case of CCM acquired in an endemic area and detected in its early stage. A 42-year-old El Salvadoran woman presented with recurrent chest pain and syncopal episodes. Significant family history includes a sister in El Salvador who also began suffering similar episodes. Physical exam and ancillary studies were only remarkable for sinus bradycardia. The patient was diagnosed with symptomatic sinus bradycardia and a pacemaker was placed. During her hospital course, Chagas serology was ordered given the epidemiological context from which she came. With no other identifiable cause, CCM was the suspected etiology. This case highlights the underrecognized presence of Chagas in the United States and the economic and public health importance of its consideration in the etiological differential diagnosis of electrocardiographic changes among Latin American immigrants. While the United States is not considered an endemic area for Chagas disease, the influx of Latin American immigrants has created a new challenge to identify at-risk populations, diagnose suspected cases, and provide adequate treatment for this disease.

## 1. Introduction

Chagas cardiomyopathy is a vector-borne parasitic infection caused by the protozoa* Trypanosoma cruzi* [1]. With the highest parasitic disease burden described in the Americas, the World Health Organization estimates that there are 5–8 million people infected with chronic Chagas disease worldwide [2, 3]. The disease is endemic in Latin America and is the leading cause of nonischemic cardiomyopathy in the region [4].

The infection is most commonly transmitted by insects of the Reduviidae family at blood meal bite sites but may also be transmitted vertically from mother to child, through blood transfusions, organ donation [5], and anecdotally in outbreaks from oral consumption of contaminated food products [6]. After the acute infection, the disease progresses to an indeterminate form, with 20–30% of individuals progressing to symptomatic cardiac manifestation of the disease after 15–25 years [1]. Chronic Chagas cardiomyopathy (CCM) most commonly presents as conduction system abnormalities, dilated cardiomyopathy, and apical aneurysms with consequent thrombus formation and embolization [7]. Chagas disease in nonischemic cardiomyopathy is associated with worse outcomes [8], and the clinical consequence of CCM can be seen in a study which found that that* T. cruzi* positive patients had a significantly higher prevalence of arrhythmias, severe heart failure, and mortality with respect to their seronegative peers [9]. Life expectancy of patients living with symptomatic CCM is less than 30% at 5 years [9].

While Chagas has traditionally been considered a disease restricted to areas of endemicity, the ever-increasing mobility of populations across regions and borders has changed disease distribution dynamics [5]. There are an estimated 300,000 people living in the United States with Chagas today [2], the vast majority of which are suspected to have acquired the disease in the endemic areas from which they emigrated.

In this paper, we present a case of Chagas cardiomyopathy acquired in an endemic area, detected in its early stages of chronic manifestation and before the classic late-stage morphologic changes to the myocardium. This case is representative of the underappreciated prevalence of the disease and its consequences in an area with a large immigrant population of Latin American origin.

## 2. Case Presentation

A 42-year-old El Salvadoran woman presented to the ED after a syncopal episode. The patient's symptoms began with left sided chest pain radiating to her left arm followed by dizziness. She had loss of consciousness for 5 minutes and had suffered multiple syncopal episodes with increasing frequency over the past year. She was advised to see a cardiologist for slow heart rate but did not follow up. She denied palpitations, vision or hearing changes, or seizure activity.

The patient grew up in rural El Salvador and immigrated to the United States 10 years ago. Of note, her sister of 40 years of age, who currently lives in El Salvador, also recently began suffering similar episodes over the past 2 years.

**Figure 1:**
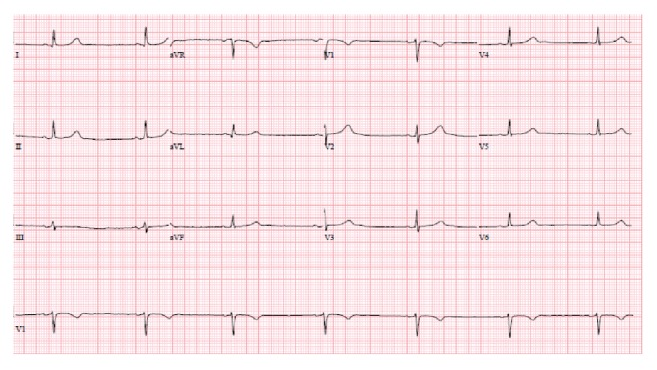
EKG of the patient on admission. This EKG shows marked sinus bradycardia with borderline low voltage QRS (QRS amplitude max: 7 mm in limb leads and 8 mm in precordial leads; HR: 41 beats/min (R-R interval: 1460 ms); PR: 146 ms; QRS 76 ms; QT/QTc 518/427 ms).

**Figure 2:**
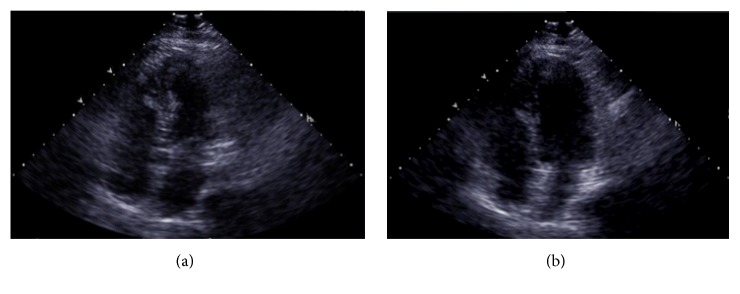
Systolic (a) and diastolic (b) apical 4 chamber transthoracic echocardiographic images. Ejection fraction estimated to be 62%. No regional wall abnormalities or valve pathology was described.

On examination, her blood pressure was 105/66 mmHg and was found to be bradycardic; her heart rate fluctuated between 30 and 42 beats per minute. The patient's lungs were clear. There was no lymphadenopathy or peripheral edema. Investigations showed negative serial troponin levels and normal hematologic and blood chemistry tests. The patient's electrocardiogram was remarkable for sinus bradycardia, with conserved axis and segment intervals and no other conduction abnormalities (Figure 1). Chest X-ray was normal. Her cardiac ultrasound showed an ejection fraction of 62% (Figure 2). The patient was diagnosed with symptomatic sinus bradycardia. A pacemaker was placed.

During her initial hospital course, a first Chagas serology test was ordered given the fact that the patient had no other identifiable causes or risk factors for cardiomyopathy beyond the epidemiological context from which she came. This result came back positive after discharge, and the patient was followed up as an outpatient for a confirmatory second serology, which was also positive. Without any other clear etiology, it is suspected that Chagas was the cause of the cardiomyopathy.

It was agreed upon by the medical team that, given the seropositive status of the patient with only electrocardiographic evidence of disease and no appreciable cardiomegaly or congestive changes, antiparasitic treatment should be offered. This issue was discussed at length in follow-up visits with the patient. While she initially expressed interest in receiving pharmacological treatment, she has been persistently hesitant to commence therapy. This remains an active discussion with the patient.

## 3. Discussion

This case highlights several key points as a result of the underrecognized prevalence of Chagas in the United States and the importance of considering Chagas in the etiological differential diagnosis of electrocardiographic changes among Latin American immigrants. Two clear issues that must be addressed from the clinical and public healthcare stewardship standpoint are the fact that the patient has 4 children who could potentially be seropositive and in the indeterminate phase and must therefore be screened and the fact that the patient has no macroevidence of CCM, and the question remains of whether or not to offer her treatment that could change the course of disease.

### 3.1. Epidemiologic Aspects

One key aspect of this case is recognizing the heterogeneity of the endemic nature of Chagas. In the case of El Salvador, the country of origin of this patient, the countrywide prevalence is 3.37% [10], third in Latin America [2]. The problem transcends in the erroneous assumption that this prevalence is equal countrywide. In the same report issued by the ministry of health of El Salvador, the area from which our patient emigrated has recent reports of seropositivity greater than 20%, largely related to the distribution of the vector and the rural environment [10]. In this sense, it is important to understand that not all immigrants can be considered under the same criteria due to the microcosm from which they come and that the sociological and geographic details of their history are of paramount importance to the adequate treatment of these patients.

Additionally, there is an inherent obligation from the part of the medical profession in the United States to maintain an index of suspicion for Chagas, as it is frequently underappreciated by the immigrant population as well, as was observed in a study in Latin American immigrants living in Los Angeles [11].

### 3.2. Recognition and Staging of Chagas Disease

Chagas disease follows a biphasic progression in which the acute phase typically culminates in a 4- to 8-week mild febrile illness and skin welt at the site of infection. This gives way to an indeterminate phase, of which 20–30% of infected persons will evolve to become symptomatic in 15–25 years with CCM [1, 12]. The earliest manifestations are alterations of the myocardial conduction system, resulting in left anterior fascicular block, right bundle branch block, bradyarrhythmias, and high grade A-V blocks, which are accompanied by symptoms like presyncope and syncope, atypical chest pain, exertional dyspnea, and palpitations [9]. When evaluating our patient across the different proposed Chagas classification criteria [13], the appearance of electrocardiography abnormalities seen in our patient with a preserved ejection fraction >45%, intermediate disease progression is implied. Additionally, parasympathetic dysautonomy has been found to exist without gross morphologic or electrocardiographic evidence of CCM and has been suspected to be involved in the higher incidence of sudden cardiac death seen in chronic seropositive Chagas patients [14]. These changes could account for the baseline regularity of the bradycardia as well as the multiple episodes of syncope and presyncope seen in our patient [15] and, along with the chronic inflammatory and autoimmune activity triggered by* T. cruzi,* are suspected to play an important part in the more commonly recognized morphologic changes of the myocardium resulting in dilated cardiomyopathy and congestive heart failure [1].

### 3.3. Treatment

While there is clear indication that treatment of Chagas disease in the acute phase has proven efficacy [16], there is still a lack of consensus with respect to the treatment of the indeterminate and chronic phases of Chagas disease. On one hand, parasite related outcomes (seroconversion) are shown to improve after treatment in the chronic phase of disease [17]. However, the change in disease course is still a matter of debate [1, 18, 19].

The only large scale trial to date showed that while treatment in patients with established CCM reduced serum parasite detection, a reduction in cardiac clinical deterioration was not appreciated over the following 5 years of follow-up [18]. However, this study has been criticized for its heterogeneity, low benznidazole dose, and lack of control of multiple confounding factors [19–22]. It also only provides definitive evidence for those with established CCM [18, 19]. There is, however, observational evidence that antitrypanosomal treatment can slow the progression of the disease. Viotti et al. showed that, in a randomized but nonblinded prospective follow-up of patients with established Chagas disease, those treated with a course of benznidazole had significantly less disease progression and mortality over an average of almost 10 years of follow-up [23]. Another recently completed longitudinal study of 310 patients with chronic Chagas disease in the indeterminate phase showed that after a mean of 19-year follow-up, the cohort treated with benznidazole had significantly less ECG changes, lower all-cause mortality, and lower cardiac related mortality after controlling for confounding cardiac risk factors [24].

In consideration of the side-effect profile of antitrypanosomal treatment, a Cochrane review found that severe side effects occurred in 2.4% of patients to benznidazole, with mild skin reactions, severe GI intolerance, lymphadenopathy, neuritis, and fever, while nifurtimox carried a 6.7% incidence and included neuritis and hepatitis [17]. Therefore, given the available evidence, current World Health Organization consensus recommends offering antitrypanosomal treatment to all patients in the indeterminate phase of the disease as long as risk and benefits are clearly explained [16], as was offered in this case.

Regarding prevention of sudden cardiac death in patients with CCM presenting with symptomatic bradyarrhythmias, placement of a pacemaker, as was done in this patient, follows best practices guidelines established in areas in which Chagas is endemic [25]. In the case of ventricular arrhythmias and significant heart failure with reduced ejection fraction, placement of an ICD in previously symptomatic individuals has been shown to have a protective effect against sudden cardiac death in the albeit limited evidence available [26, 27] and is recommended in clinical guidelines of areas of endemicity [25].

### 3.4. Vertical Transmission and Secondary Prevention

Vertical transmission of Chagas disease from a seropositive mother to offspring carries a risk of 5% [28, 29]. As outlined by Bern and Montgomery, the incidence of congenital Chagas is projected to be equivalent to that of phenylketonuria [2], a condition for which screening has already been proven to be cost-effective and has been incorporated into the prenatal screening algorithm. The WHO recommendations state that Chagas serology screening should be done in all newborns of seropositive mothers, as well as their siblings; and treatment should be offered to all children and, as previously stated, adults in the indeterminate phase [16].

An economic analysis of the cost-effectiveness of screening versus no screening done in Spain, a nonendemic area with Latin American immigrants like the United States, showed that antenatal screening of Latin American mothers, their offspring in positive cases, and consequent treatment resulted to be more cost-effective than not screening when projecting the long-term healthcare costs a seropositive person would place upon the system [30]. This is supported by an economic analysis of the annual healthcare cost burden of Chagas in the United States, which was estimated at an average of 2,162 USD annually [31]. If we take into consideration that our patient has 4 children and has an unknown time of infection with Chagas, coupled with the vertical transmission rate, it is in the best interest of the patient, her family, and the economic wellbeing of the healthcare system to realize screening serology on her children and treat accordingly if seropositive. This not only has the opportunity to improve patient outcomes but also supports significant cost-savings for the healthcare system.

### 3.5. Conclusions

While the United States is not considered an endemic area for Chagas disease, the significant influx of immigrant populations from Latin America has created a new challenge to identify susceptible populations, diagnose suspected cases, and provide adequate treatment for this disease. The opportunity to carry out appropriate population based screening and offer secondary and tertiary prevention of disease progression is emblemized by this case and is an area that offers the opportunity to improve population-directed patient care and cost-effectiveness of healthcare delivery.

Although there is inconclusive evidence regarding the etiologic treatment of the indeterminate form of Chagas disease, small studies have reported a decrease in disease advancement after treatment. This is an area that should continue to be investigated in the future. With respect to prevention of sudden cardiac death, current guidelines for pacemaker placement prove to be beneficial.

Finally, there is substantial evidence to suggest that the realization of screening to suspected cases, especially during the indeterminate form, as in the children of the index patient in this case, would lead to a substantial savings for the healthcare system.
